# Rice Gall Dwarf Virus Promotes the Propagation and Transmission of Rice Stripe Mosaic Virus by Co-infected Insect Vectors

**DOI:** 10.3389/fmicb.2022.834712

**Published:** 2022-02-11

**Authors:** Dongsheng Jia, Guozhong Luo, Wei Shi, Ye Liu, Huan Liu, Xiaofeng Zhang, Taiyun Wei

**Affiliations:** Vector-borne Virus Research Center, Fujian Province Key Laboratory of Plant Virology, Fujian Agriculture and Forestry University, Fuzhou, China

**Keywords:** rice stripe mosaic virus, rice gall dwarf virus, co-infection, *Recilia dorsalis*, synergism, transmission

## Abstract

Rice stripe mosaic virus (RSMV), a newly discovered plant cytorhabdovirus, and rice gall dwarf virus (RGDV), a plant reovirus, are transmitted by leafhopper *Recilia dorsalis* in a persistent-propagative manner. In this study, field surveys in Luoding city, Guangdong province of southern China, showed that RSMV and RGDV frequently co-infected rice plants. Furthermore, this co-infection had a synergistic effect on viral replication potential and pathogenicity in rice plants. Meanwhile, RSMV and RGDV also co-infected *R. dorsalis* vectors, and RGDV significantly promoted the propagation of RSMV in co-infected vectors. Accordingly, co-infection significantly promoted the acquisition and transmission efficiencies of RSMV by *R. dorsalis*. However, such co-infection did not significantly affect the propagation of RGDV in vectors. More importantly, we also observed that non-viruliferous *R. dorsalis* preferred to feed on co-infected rice plants, and this process further affected the feeding behavior of *R. dorsalis* to enhance viral release into rice phloem. These results provided the clues as to why RSMV had been a gradually expanding problem, creating an increasing risk of damage to rice production. Our findings revealed that synergism between RSMV and RGDV in their host and vector enhanced the propagation and transmission of RSMV, which will help guide the formulation of viral control strategies.

## Introduction

In major rice-growing countries, viral outbreaks in rice have occurred frequently and have inflicted widespread damage. From 1895 to 2021, 14 insect-borne rice viruses posed severe threats to stable rice production in many rice-growing countries (Wei et al., 2018). These rice viruses are mainly transmitted by four species of leafhoppers (*Nephotettix cincticeps*, *N. nigropictus*, *N. virescens*, and *Recilia dorsalis*) or three species of planthoppers (*Laodelphax striatellus*, *Nilaparvata lugens*, and *Sogatella furcifera*). Among them, some viruses were transmitted by the same vector insects, such as the transmission for rice stripe virus (RSV) and rice black-streaked dwarf virus (RBSDV) by *L. striatellus*, and for rice dwarf virus (RDV) and rice yellow stunt virus (RYSV) by *N. cincticeps* (Wei et al., 2018). In field, the co-infection of rice hosts by two or more viruses is common, such as southern rice black-streaked dwarf virus (SRBSDV) and rice ragged stunt virus (RRSV), rice stripe mosaic virus (RSMV) and rice gall dwarf virus (RGDV), rice tungro spherical virus (RTSV) and rice tungro bacilliform virus (RTBV), and RDV and RYSV (Sta Cruz et al., 2003; Li et al., 2014; Xia et al., 2016). Generally, rice viruses in co-infected plants exhibited synergistic severe symptoms, which can strongly influence viral transmission by insect vectors (Susi et al., 2015; Allen et al., 2019). One notable example is the synergism between SRBSDV and RRSV enhances viral acquisition by their respective vectors and increases RRSV incidence and epidemic in China (Li et al., 2014). However, the diseases caused by RSV and RBSDV rarely occur simultaneously in the field (Li et al., 2013). Currently, the mechanisms underlying the complex interaction relationship between rice viruses are still poorly understood.

In 2015, RSMV, a tentative new species in the genus *Cytorhabdovirus* that naturally infects rice, was discovered in Luoding city, Guangdong Province, China (Yang et al., 2017). Subsequently, RSMV has rapidly spread from southwestern Guangdong to Hainan, Guangxi, Jiangxi, Hunan, and Yunnan provinces. The distribution of RSMV disease is gradually expanding, and the risk of harm to rice production is increasing (Wang et al., 2021). Transmission of RSMV occurs through feeding on rice plants by the leafhopper *R. dorsalis* in a persistent-propagative manner (Yang et al., 2017). The leafhopper *N. virescens* has also been demonstrated to acquire and transmit RSMV (Zhao et al., 2019). The virus initially accumulates in epithelial cells of the filter chamber of *R. dorsalis*, from where it disseminates to the visceral muscles surrounding the filter chamber (Zhao et al., 2019). Subsequently, RSMV spreads quickly throughout the suspensory ligament to the salivary glands (Zhao et al., 2019). At this point, RSMV is able to spread from the filter chamber to the midgut, hindgut, esophagus, hemolymph, and central nervous system (Zhao et al., 2019). In the disease cycle described by Wang et al. (2021), RSMV-infected host plants and *R. dorsalis* nymphs that have overwintered can produce primary infections of spring rice.

Rice gall dwarf virus (RGDV), a member of the genus *Phytoreovirus* in the family *Reoviridae*, was first described in 1979 in Thailand (Omura et al., 1980) as the causative agent of a severe disease of rice in China and Southeast Asia (Putta et al., 1980; Fan et al., 1983). In the past 40 years, the disease caused by RGDV is always epidemic in the field of Guangdong, Hainan, and Guangxi provinces (Chen et al., 2016; Mao et al., 2019). Similar to RSMV, RGDV is also mainly transmitted by *R. dorsalis* in a persistent-propagative manner (Moriyasu et al., 2000). Usually, RGDV initially infects the filter chamber epithelium, then directly crosses the basal lamina into the visceral muscles, from where it spreads throughout the entire midgut and hindgut. Finally, RGDV spreads into the salivary glands (Zheng et al., 2015). Meanwhile, RGDV is mainly paternally transmitted vertically *via* the sperm (Mao et al., 2019). Vertical transmission can also occur by exploiting virus-induced tubules as a vehicle to overcome the transovarial transmission barrier in female leafhoppers (Liao et al., 2017). These vertical transmission manners facilitate the maintenance of RGDV during the cold seasons and can explain how RGDV epidemic could be established throughout Southern China for more than 30 years (Mao et al., 2019).

During our field surveys in Luoding city, Guangdong province of southern China, we observed that rice plants and insect vectors were frequently co-infected with RSMV and RGDV. In this study, we further investigate the disease occurrences and synergistic interaction caused by RSMV and RGDV. Our findings reveal that the synergism between RSMV and RGDV facilitates the propagation and transmission of RSMV by *R. dorsalis*. The findings presented here serve to elucidate the rapid epidemic mechanism of the newly identified RSMV in the field.

## Materials and Methods

### Insects, Viruses, and Antibodies

Non-viruliferous *R. dorsalis* individuals were collected from Guangdong Province, China, and propagated at 25 ± 3°C in the laboratory, as reported previously (Chen et al., 2016). The RSMV- or RGDV-infected rice plants were collected from Guangdong Province, China, and propagated *via* transmission by *R. dorsalis*, as reported previously (Yang et al., 2017). Polyclonal antibodies against RSMV nucleoprotein (N) and RGDV major outer capsid protein P8 were prepared as described previously (Zheng et al., 2015; Zhao et al., 2019).

### Field Investigation of Viral Infection in Rice Plants and Insect Vectors

Field surveys were performed between January 2018 and October 2021 in Luoding city, Guangdong, China. Rice samples showing dwarf, leaf mosaic or stripe symptoms were sampled and diagnosed by RT-PCR, as previously described (Chen et al., 2016; Yang et al., 2017). Briefly, total RNAs of virus-infected rice leaves were extracted using the TRIzol Kit (Invitrogen) following the manufacturer’s instructions. Reverse transcription was performed using RevertAid reverse transcriptase (Invitrogen) and the appropriate reverse primers following manufacturer’s instructions. The primer sequences used for amplifying RGDV or RSMV in this study were listed in Supplementary Table S1. Meanwhile, *R. dorsalis* individuals were collected and the viral accumulation was detected by immunofluorescence microscopy, as previously described (Zheng et al., 2015; Zhao et al., 2019). Briefly, 200 second-instar *R. dorsalis* fed on RSMV and RGDV singly or co-infected rice plants for 1 day, respectively, and then placed on healthy rice plants. At 2, 4, and 6 days post-first access to diseased plants (padp), the internal organs of 50 insects were dissected and fixed in 4% paraformaldehyde for 2 h and permeabilized in 2% Triton X-100 in .01 M PBS for 30 min at room temperature. Finally, the internal organs were incubated with RSMV-N-specific IgG conjugated directly to rhodamine (N-rhodamine) and RGDV-P8-specific IgG conjugated directly to fluorescein isothiocyanate (FITC) (P8-FITC) according to the manufacturer’s instructions (Thermo Fisher).

### RT-qPCR and Western Blot Assays of Viral Loads in Rice Plants and Insect Vectors

To understand the synergistic interaction of RSMV and RGDV in rice, singly or co-infected *R. dorsalis* individuals were fed on rice seedlings at the third leaf stage for 2 days, respectively. Then, the rice seedlings were planted in the field and were screened by RT-PCR assay at 30 days post-inoculation (dpi). Virus-positive samples were defined as diseased rice plants. Total RNAs were isolated from the infected leaves to quantify viral loads of RSMV or RGDV by RT-qPCR assay. The RT-qPCR assay was performed in triplicate using the SYBR Green PCR Master Mix kit (Promega, United States) according to the manufacturer’s instructions. The transcript level of actin was served as the control. Relative gene expression levels were detected using the 2^−ΔΔCT^ method (Livak and Schmittgen, 2001). To understand the synergistic interaction of RSMV and RGDV in insects, second-instar nymphs of *R. dorsalis* were allowed to feed on RSMV and RGDV singly or co-infected tillering stage rice plants (*Oryza sativa* L. cv. “Nipponbare”) for 1 day and then placed on healthy rice seedlings. At 2, 4, and 6 days padp, total RNAs were isolated from 30 insect vectors to quantify viral loads of RSMV or RGDV by RT-qPCR assay as described above.

To determine the accumulation levels of viral protein in rice and insects, total proteins from three virus-positive rice seedlings or 50 intact insects were extracted and separated by SDS-PAGE. Then, the accumulation levels of viral proteins were detected by western blot assay using RGDV-P8-specific IgG or RSMV-N-specific IgG. The expression level of actin was served as the control using Actin-specific IgG (Sigma). The proteins were visualized with the Luminata Classico Western HRP Substrate (Millipore) and imaged with the Molecular Imager ChemiDoc XRS+ System (Bio-Rad), as described previously (Chen et al., 2017b).

### Acquisition and Transmission Rates of RSMV or RGDV by *Recilia dorsalis*

To investigate the acquisition and transmission rates of viral co-infection by *R. dorsalis*, 200 s-instar insects were fed on RSMV-infected, RGDV-infected, or co-infected rice plants for 2 days. Then, these insects were placed on healthy rice plants at 16 h light/8 h dark and 70% relative humidity. At 10 days post-first access to diseased plants (padp), *R. dorsalis* individuals were placed in glass tubes that contained a single rice seedling and then were kept for 10 days with seedlings replaced daily, as described previously (Chen et al., 2016). The test was conducted using 30 *R. dorsalis* individuals and three replicates. The insects were collected, and virus loads were assessed by RT-PCR assay at 20 days padp. The plants inoculated with confirmed viruliferous insects were subjected to RT-PCR assay 10 days later. The transmission rate of RSMV or RGDV by *R. dorsalis* was calculated.

### Electron Microscopy

The rice leaves (healthy, RSMV-infected, RGDV-infected, or co-infected) or the intestines from *R. dorsalis* vectors (non-viruliferous, RSMV-infected, RGDV-infected, or co-infected) were fixed, dehydrated, embedded, and thin sections were cut, as previously described (Wei et al., 2006). Sections were observed with a transmission electron microscope (H-7650, HITACHI).

### Plant Selection Preference Assay

We then clarify the effects of rice plants infected with different viruses on *R. dorsalis* feeding tendency. A glass Y-tube olfactometer was applied to test plant odor selection preference of *R. dorsalis* following the method described by Signoretti et al. (2012) and Yi et al. (2021), with minor modifications. Briefly, The Y-shaped tube (4-cm inner diameter) comprised a central tube (10 cm long) and two arms (18 cm long, offset by 75°) connected to bags containing a living plant each. The airflow was guided through an activated carbon purifier and a distillation water bottle before entering the odor source to purify the air and increase the humidity. A flow meter was used to calibrate the airflow in the olfactometer (500 ml/min) at the end of each arm. The Y-tube was horizontally positioned in an airtight cubic box by placing a 30-W filament lamp 30 cm above it. Thirty *R. dorsalis* adults (starved for 2 h before testing) were placed in the central tube and observed for 3 h in each experimental group. Each experiment was repeated three times. The preference of non-viruliferous *R. dorsalis* adults to healthy, RGDV-infected, RSMV-infected, or co-infected rice plants was assessed.

### Electrical Penetration Graph Recordings

We then used EPG analysis to investigate the effects of viral co-infection on insect feeding behavior. The EPG records were obtained using a DC-monitor, GIGA-8 model as described previously (Yi et al., 2021). Briefly, the healthy, RSMV-infected, RGDV-infected, or co-infected rice seedlings were planted into soil-filled plastic pots. The substrate voltage electrode (*D* = 2 mm, *H* = 10 cm) was inserted into the soil of the plastic pots, and the substrate voltage was adjusted so that the EPG signals ranged from −5 to +5 V. The non-viruliferous adult *R. dorsalis* were starved for 1 h, transferred to glass tubes and then put on CO_2_ under anesthesia for 10 s. The dorsal thorax of *R. dorsalis* was immobilized to one end of a gold wire electrode (*D* = 20 μm, *H* = 10–15 cm) with water-soluble silver glue. The other end of the gold wire electrode was connected to a copper extension wire. The wired insect was connected to the EPG probe *via* a copper nail. The probe was connected to the amplifier, and the insect was placed on the rice leaf. A copper wire (*D* = 2 mm, *H* = 10 cm) was inserted into the potting soil vertically and connected to another amplifier. Subsequently, the electrical EPG signals were amplified and digitized using a converter. The plant, insect, and amplifiers were placed inside a Faraday cage, to cancel any external noise sources, in a temperature-controlled laboratory at 28 ± 2°C. The STYLET 3.8 software (Tjallingii and Hogen Esch, 1993) was used for data acquisition. Data for each *R. dorsalis* were continuously recorded for 5 h using the random design method. At least 20 independent biological replicates were conducted for each rice seedling.

### Statistical Analyses

All data were analyzed with SPSS, version 17.0. Multiple comparisons of the means were conducted using a Tukey’s honest significant difference (HSD) test with a one-way ANOVA. The comparisons of the means in plant selection preference assay were conducted using a contingency table analyses with a chi-square test. The data were uniformly back-transformed after analysis for presentation in the text and figures.

## Results

### Rice Plants Co-infected With RSMV and RGDV Showed the Synergistic Symptoms

**Figure 1:**
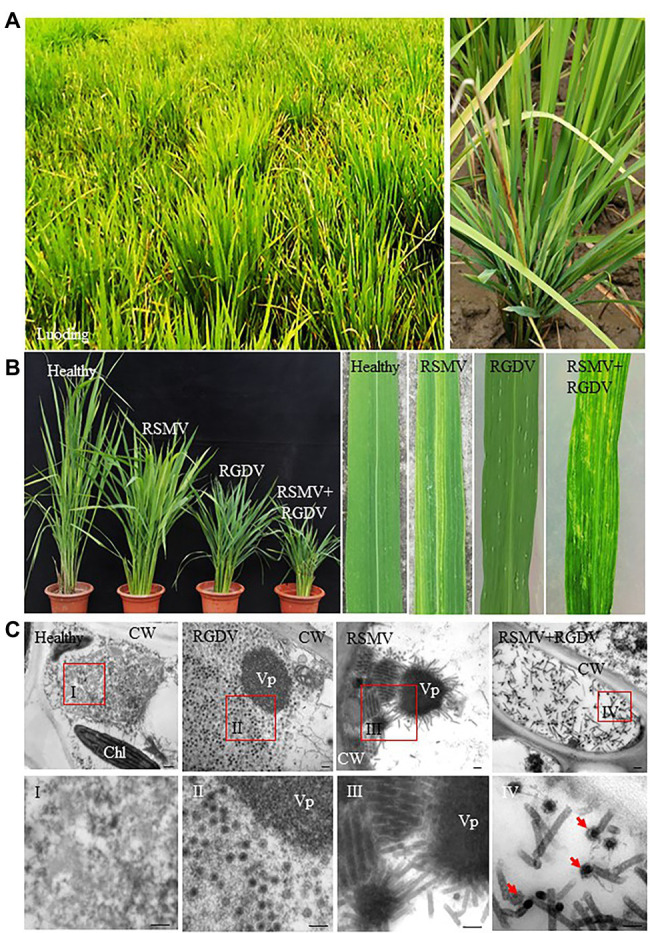
Typical signs of Rice stripe mosaic virus (RSMV) and rice gall dwarf virus (RGDV) co-infection in rice. **(A)** Symptoms of virus-infected rice in the field (Luoding, Guangdong, China). **(B)** The dwarf and mosaic signs displayed on rice plants singly infected with RSMV, RGDV or co-infected with RSMV and RGDV. **(C)** Electron micrographs of virions in RSMV-infected, RGDV-infected, or co-infected rice leaves. Healthy rice leaf was as control. Panels I–IV are the enlarged images of the red boxed areas I–IV in the above panels. Red arrows indicate a RGDV virion adhered to a RSMV virion in the cytoplasm of co-infected rice leaf. CW, cell wall; Chl, chloroplast; and Vp, viroplasm. Bars, 100 nm.

From January 2018 through October 2021, rice viral diseases in the field of Luoding city were assessed, and results showed that most of rice viral diseases were caused by the co-infection with RSMV and RGDV. The rice plants singly infected with RSMV exhibited obvious yellow stripes and mosaic symptoms, the rice plants singly infected with RGDV exhibited dwarfing with abundant galls along the leaf sheaths, while the rice plants co-infected with RSMV and RGDV exhibited severe dwarfing, mosaic and galls along the leaf sheaths (Figures 1A,B). We randomly collected rice plants with mosaic stripe or gall dwarf symptom and calculated that about 5%–20% or 9%–27% of rice plants were single RSMV- or RGDV-positive, respectively; however, 55%–84% of rice plants were co-infected (Figures 1A,B; Table 1). Furthermore, more than 69% of virus-infected overwintered rice plants were co-infected (Table 1), which became the source of viruses to subsequently infect overwintered *R. dorsalis* to continue the disease cycle in spring. Electron micrographs showed that the bacilliform virions of RSMV and spherical virions of RGDV were orderly distributed in the cytoplasm of singly infected parenchyma of rice phloem (Figure 1C). In co-infected regions, both virions were observed to associate together (Figure 1C). These results suggest that RSMV and RGDV may have synergistic relationship during viral co-infection of rice plants.

**Table 1:** RSMV and RGDV distribution in Luoding, Guangdong province during 2018–2021.

Sampling time	Planting time	Incidence rates in field (%)	No. of rice samples tested	Only RSMV-positive		Only RGDV-positive		Both RSMV- and RGDV-positive	
				No.	Rate (%)	No.	Rate (%)	No.	Rate (%)
2018/01	Overwintering rice	5–50	52	3	5.8	5	9.6	44	84.6
2018/05	Spring rice	5–30	63	9	14.3	12	19.0	42	66.7
2018/10	Late summer rice	10–40	54	12	22.2	8	14.8	34	63.0
2018/12	Overwintering rice	10–45	60	6	10.0	10	16.7	44	73.3
2019/05	Spring rice	5–25	55	10	18.2	15	27.3	30	54.5
2019/10	Late summer rice	5–40	67	7	10.4	10	14.9	50	74.6
2019/12	Overwintering rice	10–40	46	8	17.4	6	13.0	32	69.6
2020/10	Late summer rice	5–55	57	5	8.8	7	12.3	45	78.9
2021/10	Late summer rice	10–75	50	7	14.0	5	10.0	38	76.0

### Viral Loads of RGDV and RSMV Were Increased in Co-infected Rice Plants

**Figure 2:**
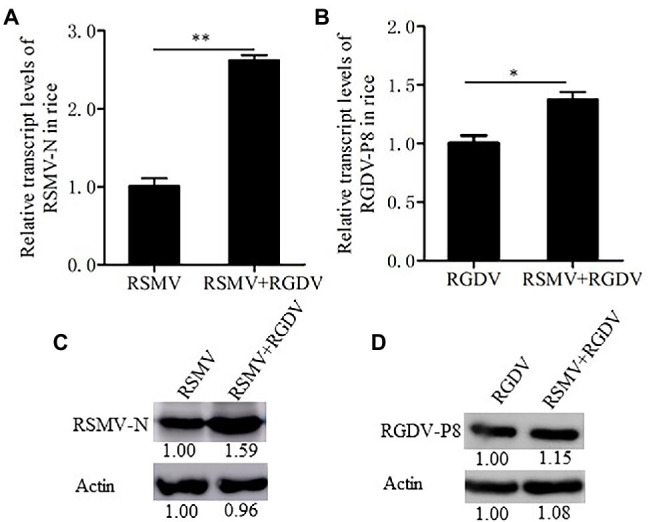
Co-infection with RSMV and RGDV significantly increased the accumulation of RSMV in rice plants. **(A)** The relative transcript levels of RSMV N were determined by RT-qPCR at 30 dpi in rice plants infected with either RSMV or both RSMV and RGDV. **(B)** The relative transcript levels of RGDV P8 were determined by RT-qPCR at 30 dpi in rice plants infected with either RGDV or both RSMV and RGDV. Means (±SD) from three biological replicates are presented. ^*^p < .05 and ^**^p < .01. **(C,D)** The protein accumulation levels of RSMV N **(C)** or RGDV P8 **(D)** in rice plants singly infected with RSMV, RGDV or co-infected with RSMV and RGDV at 30 dpi were determined by Western blots. Plant actin was detected as a control.

To understand whether RSMV and RGDV have synergistic interaction in rice plants, viral loads of RSMV and RGDV in single or co-infected rice plants were measured by RT-qPCR assay. At 30 dpi, RSMV loads in co-infected rice plants were 2.5 times higher than that in RSMV singly infected rice plants, while RGDV loads in co-infected rice plants were 1.4 times higher than that in RGDV singly infected rice plants (Figures 2A,B). The accumulation levels of RSMV N and RGDV P8 were quantified by western blot assay. As a result, the RSMV N accumulated to higher level in co-infected leaves than in RSMV singly infected rice plants (Figure 2C). However, the accumulation levels of RGDV P8 in co-infected leaves similarly with that in RGDV singly infected leaves (Figure 2D). These results suggest that the co-infection significantly increased the viral production of RSMV more than that of RGDV in rice plants.

### Co-infection of *R. dorsalis* With RSMV and RGDV Showed the Synergistic Relationship

As the common vector of RSMV and RGDV, *R. dorsalis* may become naturally infected with both RSMV and RGDV. Analysis of samples collected during outbreaks in rice plants in Luoding showed that 6%–12% of insects were single RSMV-positive, 8%–14% were single RGDV-positive, while 20%–36% were positive for both RGDV and RSMV (Table 2). Interestingly, virus-positive rates of *R. dorsalis* during the overwinter season remained at 38%–46% (Table 2). This observation suggests that the viruses overwinter in viruliferous insects, which creates a likelihood of transmission to gramineous weeds or new rice seedlings in the early spring season. These results suggest that RSMV and RGDV may have synergistic relationship during viral infection of insect vectors in the diseased field.

**Table 2:** The rates of RSMV- or RGDV-positive *Recilia dorsalis* from diseased field in Luoding, Guangdong province during 2018–2020.

Sampling time	Planting time	No. of insects tested	Only RSMV-positive		Only RGDV-positive		Both RSMV- and RGDV-positive	
			No.	Rate (%)	No.	Rate (%)	No.	Rate (%)
2018/01	Overwintering rice	100	10	10	8	8	20	20
2018/05	Spring rice	100	6	6	12	12	30	30
2018/10	Late summer rice	100	6	6	8	8	36	36
2019/01	Overwintering rice	100	12	12	10	10	24	24
2019/05	Spring rice	100	6	6	14	14	28	28
2019/10	Late summer rice	100	10	10	10	10	34	34
2019/12	Overwintering rice	100	12	12	12	12	30	30
2020/10	Late summer rice	100	6	10	10	10	26	26

### RGDV Promoted the Propagation and Spread of RSMV in Co-infected *R. dorsalis*

**Figure 3:**
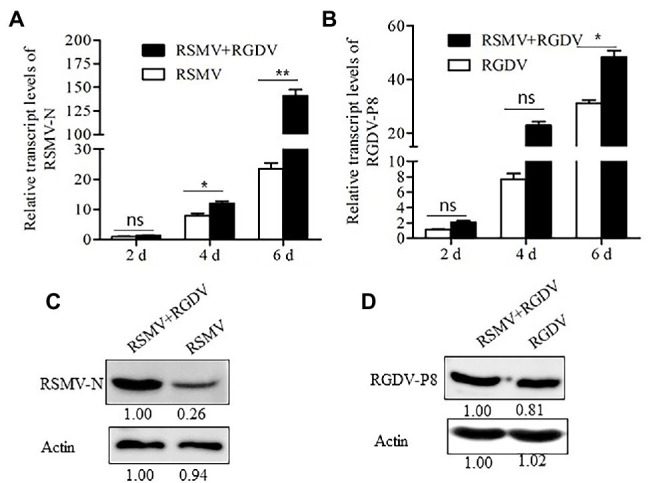
Co-infection with RSMV and RGDV significantly promoted the infection of RSMV in the *Recilia dorsalis* vector. **(A)** The relative transcript levels of RSMV N in *R. dorsalis* infected with RSMV or co-infected with RGDV and RSMV were determined by RT-qPCR at 2, 4, and 6 days padp. **(B)** The relative transcript levels of RGDV P8 in *R. dorsalis* infected with RGDV or co-infected with RSMV and RGDV were determined by RT-qPCR at 2, 4, and 6 days padp. Means (±SD) from three biological replicates are presented. ^*^p < .05; ^**^p < .01; and ns, not significant. **(C)** The accumulation levels of RSMV N in *R. dorsalis* infected with RSMV or co-infected with RGDV and RSMV for 6 days padp were detected by Western blot assay. Insect actin was detected as a control. **(D)** The accumulation of RGDV P8 in *R. dorsalis* infected with RGDV or co-infected with RSMV and RGDV for 6 days padp were detected by Western blot assay. Insect actin was detected as a control.

To understand whether RSMV and RGDV have synergistic interaction in insect vectors, 50 *R. dorsalis* individuals were fed on singly or co-infected rice plants for 1 day and then fed on healthy rice seedlings. At 2, 4, and 6 days padp, the loads of RSMV or RGDV were detected by RT-qPCR assay. The results showed that RSMV loads in co-infected insects were six times higher than that in singly infected controls at 6 days padp (Figure 3A). However, RGDV loads between co-infected and single RGDV-infected insects were not significantly different at 2 and 4 days padp (Figure 3B). However, RGDV loads in co-infected insects were approximately 1.4 times of that in the singly infected controls at 6 days padp (Figure 3B). Western blot analysis confirmed that co-infection promoted the accumulation of RSMV N, but not RGDV P8, in viruliferous insects (Figures 3C,D).

**Figure 4:**
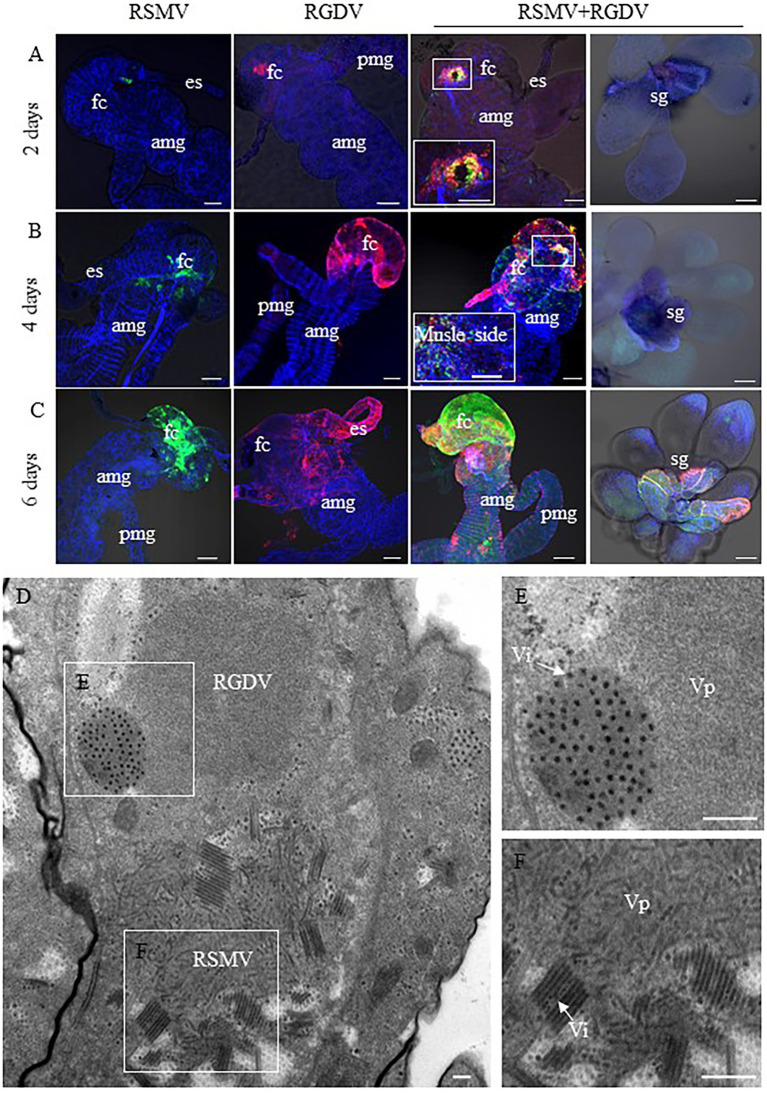
Spread of RSMV and RGDV throughout *R. dorsalis*. **(A–C)** At 2 **(A)**, 4 **(B)**, and 6 **(C)** days padp, the internal organs or salivary glands of *R. dorsalis* singly infected with RSMV, RGDV or co-infected with RSMV and RGDV were stained with RSMV N–FITC (green), RGDV Pns11-rhodamine (red) and phalloidin–Alexa Fluor 647 carboxylic acid (blue) for examination by confocal microscopy. AMG, anterior midgut; ES, esophagus; FC, filter chamber; HG, hindgut; posterior midgut; and SG, salivary glands. Bar = 100 μm. **(D)** Electron micrographs of virus in midgut of *R. dorsalis* co-infected with RSMV and RGDV. Panels **(E,F)** are enlargements of the boxed areas in panel **(D)**. Vp, viroplasm; Vi, viron. Bars = 100 nm.

Immunofluorescence microscopy was then used determine the distribution of viruses in the internal organs of co-infected *R. dorsalis*. At 2 days pdap, RSMV and RGDV only infected the initially infected epithelial cells of the filter chamber in singly infected insects. Interestingly, the co-localization of RSMV and RGDV in the same initially infected epithelial cells could be observed in co-infected insects (Figure 4A), suggesting that these two viruses could simultaneously and cooperatively enter the intestinal epithelial cells during the acquisition of viruses from co-infected rice plants by *R. dorsalis*. At 4 days pdap, RSMV started to spread to the neighboring epithelial cells from the initially infected sites of the filter chamber, while RGDV had spread to the epithelial cells of the entire filter chamber (Figure 4B). Interestingly, in co-infected insects, RSMV and RGDV were together released from the epithelial cells of the infected filter chamber to the visceral muscle tissues, but had not yet spread to salivary glands (Figure 4B). At 6 days pdap, RSMV infected the whole filter chamber, while RGDV had spread from the filter chamber to the midgut muscle tissue. In co-infected insects, both RSMV and RGDV had infected the filter chamber and midgut and then rapidly spread to salivary glands (Figure 4C). Electron microscopy showed that both RSMV and RGDV could form viroplasms to assemble progeny virions in the same midgut epithelial cell in co-infected *R. dorsalis* (Figures 4D–F). These results indicated that RSMV and RGDV could co-infect the same epithelial cell, and RGDV appeared to enhance the replication and spread of RSMV in *R. dorsalis*.

### Co-infection Improved the Acquisition and Transmission Efficiencies of RSMV in *R. dorsalis*

**Figure 5:**
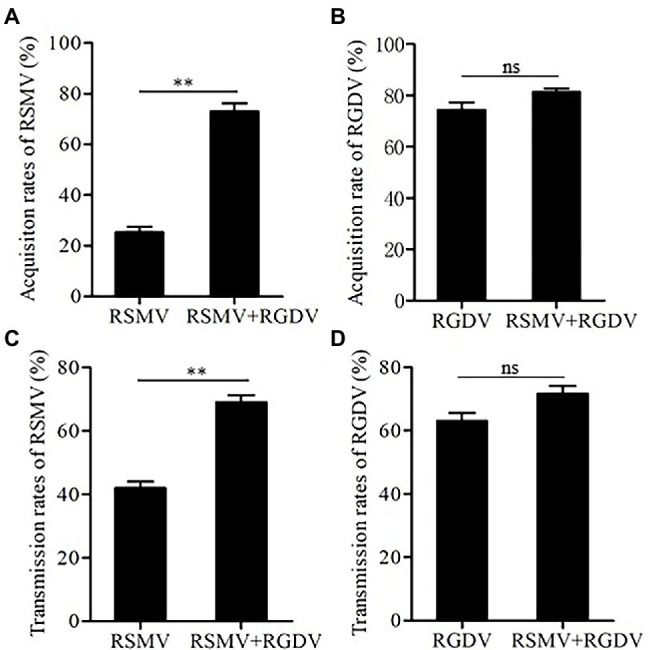
The acquisition and transmission rates of RSMV and RGDV by *R. dorsalis*. **(A)** The acquisition rates of RSMV by *R. dorsalis* fed on rice plants singly infected with RSMV or co-infected with RSMV and RGDV for 2 days. **(B)** The acquisition rates of RGDV by *R. dorsalis* fed on rice plants singly infected with RGDV or co-infected with RSMV and RGDV for 2 days. **(C)** The transmission rates of RSMV by *R. dorsalis* singly infected with RSMV or co-infected with RSMV and RGDV. **(D)** The transmission rates of RGDV by *R. dorsalis* singly infected with RGDV or co-infected with RSMV and RGDV. Means (±SD) from three biological replicates are shown. ^**^p < .01. ns, not significant.

To explore how mixed infection affected the acquisition and transmission of RSMV or RGDV by *R. dorsalis*, the virus acquisition efficiency of *R. dorsalis* from singly or co-infected rice plants was compared. It was observed that 30% and 75% of insects acquired RSMV from RSMV singly infected and co-infected rice plants, respectively (Figure 5A). In contrast, 76% and 80% *R. dorsalis* acquired RGDV from RGDV singly infected and co-infected rice plants, respectively (Figure 5B). These results suggest that co-infected rice plants improved the acquisition ability of RSMV, but not RGDV by *R. dorsalis*. Subsequently, viral transmission efficiency by singly or co-infected *R. dorsalis* was compared. The results showed that RSMV transmission efficiency by co-infected *R. dorsalis* was about 70%, which is significantly higher than about 40% observed for RSMV singly infected controls (Figure 5C). Meanwhile, RGDV transmission efficiencies by co- and singly infected *R. dorsalis* were 72% and 64%, respectively (Figure 5D). These results suggest that RGDV could enhance the acquisition and transmission efficiencies of RSMV by co-infected *R. dorsalis*.

### Effects of Co-infection on Feeding Tendency and Behavior of *R. dorsalis*

**Figure 6:**
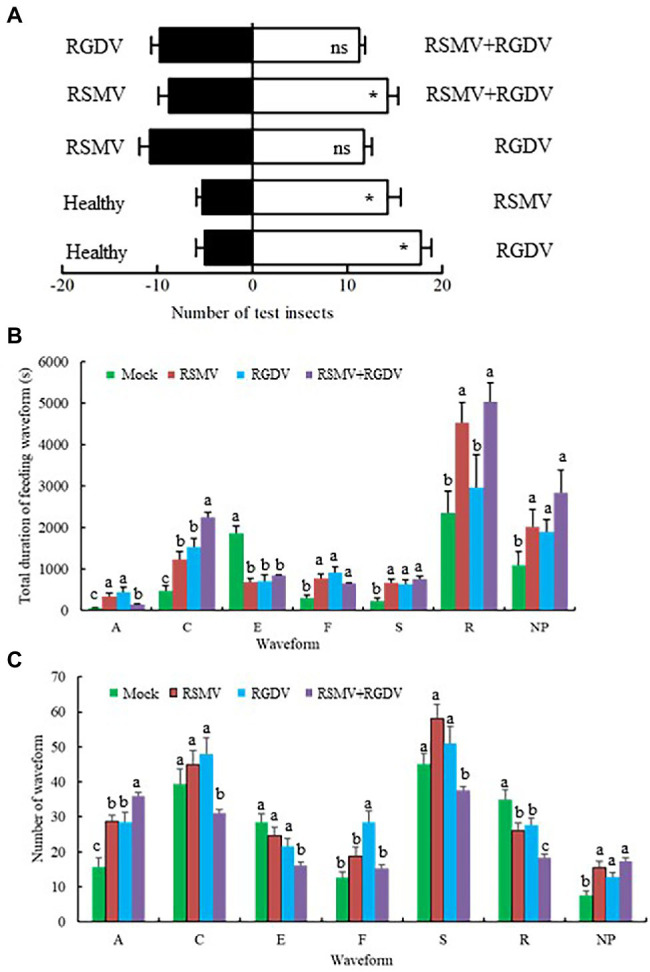
Virus-infected rice plants affected the feeding tendencies and behaviors of non-viruliferous *R. dorsalis*. **(A)** The effect of odors emitted by virus-infected rice plants on the feeding tendencies of *R. dorsalis*. Each experimental group was comprised of 30 adults and was repeated five times in total. Means (±SD) from five biological replicates are presented. ^*^p < .05; ns, not significant. **(B,C)** Virus-infected rice plants affected the feeding behaviors of non-viruliferous *R. dorsalis*. The duration of each waveform **(B)** and number of each waveform **(C)** for *R. dorsalis* were analyzed. NP, not probing; A, stylet movement in the tissue; S, intracellular salivation in mesophyll cells; C, active ingestion of the phloem; E, passive phloem sap ingestion; F, obstacle waveform; R, rest waveform; means (±SD) from three biological replicates are presented. Different letters in the same column indicate a significant difference (*p* < .05).

To clarify the effects of co-infection on feeding tendencies of vectors, the preferences of non-viruliferous *R. dorsalis* on healthy, RSMV-, RGDV-, or co-infected rice plants were compared. In general, the non-viruliferous *R. dorsalis* tended to feed on virus-infected rice plants over healthy controls (Figure 6A). When tested for the preference between the RGDV-infected and RSMV-infected or co-infected rice plants, no significant quantitative difference was observed (Figure 6A). However, *R. dorsalis* exhibited a significant tendency to feed on co-infected over RSMV-infected rice plants (Figure 6A). These results indicate that viral infection improves the attractiveness of rice plants to non-viruliferous *R. dorsalis*. More importantly, the presence of RGDV in co-infected rice plants appears to benefit RSMV transmission in field.

To clarify the effects of co-infection on feeding behavior of vectors, EPG analysis was used to compare the differences in feeding behavior of non-viruliferous *R. dorsalis* on healthy, RSMV-, RGDV-, or co-infected rice plants. *R. dorsalis* adults feeding produced seven distinct types of EPG waveforms, such as not probing (NP), stylets movement in the tissue (A), intracellular salivation in mesophyll cells (S), active ingestion from the phloem (C), passive phloem sap ingestions (E), obstacle waveform (F), and rest waveform (R). It was observed that the duration of waveform C on co-infected rice plants was significantly higher than that of other groups. The number of waveform A on co-infected rice plants was significantly higher than that of the other groups, and the number of waveform R on co-infected rice plants was significantly lower than that of the other groups (Figures 6B,C). These changes of waveform duration or frequency are expected to be beneficial to viral transmission to *R. dorsalis*.

## Discussion

Plant viruses co-infecting the same hosts may display a great variety of pathways resulting in synergistic or antagonistic interactions (García-Cano et al., 2006; Syller, 2012; Li et al., 2014). In this study, two viruses from unrelated families, *Reoviridae* and *Rhabdoviridae*, form the synergistic relationship during co-infection of rice plants and their same vector *R. dorsalis*. The disease caused by RGDV has been epidemic in the field of southern China in the past 40 years; however, the disease caused by RSMV was initially observed in the field of southern China 6 years ago. From 2015 to now, RSMV disease distribution is gradually expanding from Guangdong province to the five neighboring provinces (Wang et al., 2021). Our survey shows that RSMV and RGDV co-infection is common in the field, even up to 84%, which would be a key factor to facilitate the incidence and spread of RSMV epidemics in the field. We further show that the synergism between RGDV and RSMV enhances the acquisition and propagation of RSMV in *R. dorsalis* and thus facilitates the transmission of RSMV. In addition, non-viruliferous *R. dorsalis* prefers to feed on co-infected rice plants, and this process further affects the feeding behavior of *R. dorsalis* to enhance viral release into rice phloem. These results provide the clues as to why RSMV had been gradually expanding and rapid epidemic of RSMV disease in the field.

We observe that RSMV and RGDV could simultaneously enter the same epithelial cells of intestines during the acquisition of viruses from co-infected rice plants by *R. dorsalis*. Electron microscopy confirms that the direct association of these two types of viral particles in the parenchyma of rice phloem. Then, these two viruses assemble progeny virions in the same epithelial cells, which then together spread to the visceral muscle tissues. Our results thus reveal that RSMV and RGDV have formed the cooperative interactions during co-infection of rice hosts and insect vectors. How RGDV benefits RSMV propagation is still unknown. Previously, we have shown that the persistent infection of RGDV in *R. dorsalis* can trigger a strong small interfering RNA (siRNA) antiviral immune response to efficiently control RGDV replication below a putative cytopathogenic threshold (Lan et al., 2016). The non-structural protein Pns11 of RGDV is viral suppressor of RNA silencing (VSRs) in plant hosts (Liu et al., 2008). The highly efficient propagation of RGDV in *R. dorsalis* likely requires a balance against VSR potency and the antiviral response of the insect vector. From this point, we deduce that RGDV might encode a strong VSR for minimizing the siRNA antiviral pathway to facilitate the highly efficient propagation of RSMV in co-infected *R. dorsalis*. Furthermore, another two types of antiviral immune responses, autophagy and apoptosis, have also been triggered by RGDV to facilitate viral efficient spread in *R. dorsalis* (Chen et al., 2017a, 2019). Thus, RGDV can remodel autophagy and apoptosis responses to benefit viral propagation, which may also explain why RSMV becomes highly efficient competence with co-infected *R. dorsalis*.

In summary, we demonstrate that a rice reovirus is able to improve the transmission of another rice cytorhabdovirus by the same vector. What we have known is that RTBV (a double-stranded circular DNA virus) is dependent on RTSV (a single-stranded RNA virus) for joint transmission by leafhopper vector *N. virescenes* (Sta Cruz et al., 2003; Srilatha et al., 2019). By contrast, co-infection with a wheat rhabdovirus inhibits the acquisition and propagation of Mal de Río Cuarto virus, a plant reovirus, in their planthopper vector (Dumón et al., 2017). Similarly, RBSDV limits RSV acquisition and transmission by their planthopper vector (Moya Fernández et al., 2021). Therefore, a better understanding of how different plant viruses reciprocally affect the acquisition, propagation, and accumulation by the same insect vectors will help dissect the underlying causes of the epidemics of these viruses.

## Data Availability Statement

The original contributions presented in the study are included in the article/Supplementary Material; further inquiries can be directed to the corresponding author.

## Author Contributions

TW and DJ designed all experiments, analyzed the data, organized the project, and wrote the manuscript. DJ, GL, WS, and XZ performed the field survey. DJ, YL, and HL performed the experiments for immunofluorescence staining and electron microscopy. All the authors contributed to the article and approved the submitted version.

## Funding

This project was supported by funds from the National Natural Science Foundation of China (31920103014 and 31970160), the Natural Science Foundation of Fujian Province (2020J06015 and 2019J01373), and the Science, Technology and Innovation Foundation of Fujian Agriculture and Forestry University (CXZX2019004K and CXZX2019021G).

## Conflict of Interest

The authors declare that the research was conducted in the absence of any commercial or financial relationships that could be construed as a potential conflict of interest.

## Publisher’s Note

All claims expressed in this article are solely those of the authors and do not necessarily represent those of their affiliated organizations, or those of the publisher, the editors and the reviewers. Any product that may be evaluated in this article, or claim that may be made by its manufacturer, is not guaranteed or endorsed by the publisher.
