# Supplementary material for: Rice Gall Dwarf Virus Promotes the Propagation and Transmission of Rice Stripe Mosaic Virus by Co-infected Insect Vectors
Source: Front Microbiol. 2022 Feb 11;13:834712. doi: 10.3389/fmicb.2022.834712 (PMC8874222; doi:10.3389/fmicb.2022.834712)
Supplement: Supplementary file 1 [file Table_1.doc]

S1 Table Primers used in this study.

| Primer name | Primer sequences | |
| --- | --- | --- |
|  | Forward | Reverse |
| RGDV-P8 | 5'- ATTTCGGAGTATGGGACC-3' | 5'- CCAGGGTGATACAAAAGC -3' |
| RSMV-N | 5'- ATGGCAACCGACAAGTCTTTTG-3' | 5-' CCTCGGCTGTAGATTGATCCA -3' |
| q-RGDV-P8 | 5'- GATTCAAGGGGCACAGAACG-3' | 5'- GTAATGGTTGCGACTGGGTC- 3' |
| q-RSMV-N | 5'- CCGATTACCTGCCTAAGACAACA-3' | 5'- GCCTTTTTAGTATCCGCATCAGT - 3' |
| q-eEF-1 | 5'-CAGTGAGAGCCGTTTTGAG-3' | 5'-AGGGCATCTTGTCAGAGGGC- 3' |
